# Laparoscopic retrograde (fundus first) cholecystectomy

**DOI:** 10.1186/1471-2482-9-19

**Published:** 2009-12-11

**Authors:** Michael D Kelly

**Affiliations:** 1 Department of Upper GI Surgery, Frenchay Hospital, Bristol, UK

## Abstract

**Background:**

Retrograde ("fundus first") dissection is frequently used in open cholecystectomy and although feasible in laparoscopic cholecystectomy (LC) it has not been widely practiced. LC is most simply carried out using antegrade dissection with a grasper to provide cephalad fundic traction. A series is presented to investigate the place of retrograde dissection in the hands of an experienced laparoscopic surgeon using modern instrumentation.

**Methods:**

A prospective record of all LCs carried out by an experienced laparoscopic surgeon following his appointment in Bristol in 2004 was examined. Retrograde dissection was resorted to when difficulties were encountered with exposure and/or dissection of Calot's triangle.

**Results:**

1041 LCs were carried out including 148 (14%) emergency operations and 131 (13%) associated bile duct explorations. There were no bile duct injuries although conversion to open operation was required in six patients (0.6%). Retrograde LC was attempted successfully in 11 patients (1.1%). The age ranged from 28 to 80 years (mean 61) and there were 7 males. Indications were; fibrous, contracted gallbladder 7, Mirizzi syndrome 2 and severe kyphosis 2. Operative photographs are included to show the type of case where it was needed and the technique used. Postoperative stay was 1/2 to 5 days (mean 2.2) with no delayed sequelae on followup. Histopathology showed; chronic cholecystitis 7, xanthogranulomatous cholecystitis 3 and acute necrotising cholecystitis 1.

**Conclusions:**

In this series, retrograde laparoscopic dissection was necessary in 1.1% of LCs and a liver retractor was needed in 9 of the 11 cases. This technique does have a place and should be in the armamentarium of the laparoscopic surgeon.

## Background

**Figure 1:**
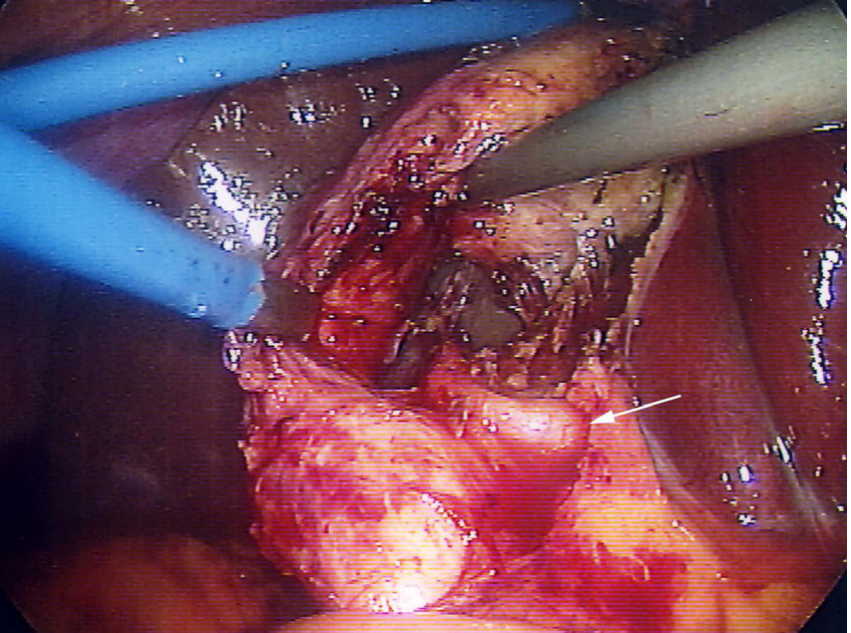
**Laparoscopic view of standard dissection technique in a Mirizzi type I arrangement**. Fundic traction gave good exposure of Calot's triangle and there was no need for retrograde (fundus first) dissection. Accurate transcystic cholangiography would, however, have been very difficult. Arrow points to the right hepatic artery crossing the common hepatic duct.

Problems with laparoscopic cholecystectomy (LC) include bile duct injury (BDI), conversion or failure to convert to open operation. The standard technique uses a grasper on the fundus of the gallbladder to apply cephalad traction to elevate the liver to expose Calot's triangle for dissection (figure 1). While a rapid, simple and proven technique this manoeuvre does lead to distortion of the biliary anatomy [1]. Hunter brought attention to the importance of lateral traction on the neck of the gallbladder to open out Calot's triangle and Strasberg's writings on the "critical view of safety" have been influential [2,3]. Intraoperative cholangiography, use of 30° laparoscope and extrabiliary reference points may play some role in avoiding BDI and allowing a safer LC [4,5].

**Figure 2:**
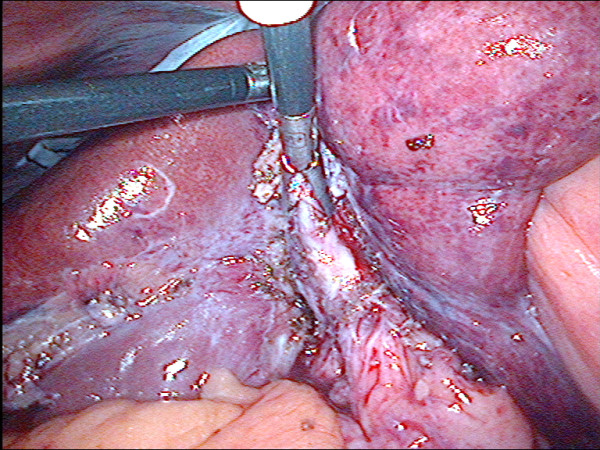
**Laparoscopic view showing failure of standard technique**. There is poor exposure and inability to safely dissect Calot's triangle with standard cephalad fundic traction (case 1).

There are some cases where standard retraction fails to expose Calot's triangle or allow safe dissection and this usually results in conversion to open operation (figure 2). A low threshold for conversion is generally considered to be a marker of good practice, however conversion is associated with increased costs and both short and long term morbidity [6]. In the era of open surgery, retrograde or "fundus first" dissection was used routinely by many surgeons while others reserved it as a defensive technique for the difficult case. When a LC is converted to an open operation, retrograde dissection is generally used [7]. Retrograde laparoscopic cholecystectomy (RLC) appears to have been underutilized possibly because in the early days of LC only rudimentary instrumentation was available. However, laparoscopic liver retractors are now readily available and the gallbladder can be mobilised fundus first whilst the liver is kept elevated by a retractor. Despite this even relatively recent influential articles have stated that the fundus first technique is difficult to apply in LC because of loss of traction on the liver when the fundus is mobilized [5].

While RLC is feasible it is not widely practiced and its true role would seem, as yet, to be undefined. Therefore an analysis of unselected LCs carried out by an experienced laparoscopic surgeon using modern instrumentation seems worthwhile.

## Methods

Following my appointment in Bristol in 2004 a prospective record was kept of a personal series of 1041 consecutive LCs carried out in the National Health Service. This was a slightly unusual group of patients as it included a large number with advanced pathology as in this period extra funds were made available to reduce the long cholecystectomy waiting times.

LC was done using standard technique with 3 or 4 ports, electrocautery and a 30° laparoscope. Early on in the series only selected cases had imaging of the bile duct but this selective policy was soon replaced by routine laparoscopic ultrasound or operative cholangiography. The initial step was to place a grasper on the fundus of the gallbladder and elevate the liver to expose Calot's triangle. Sometimes extra manoeuvres were needed to allow full exposure such as placement of an extra port or use of a fan retractor to retract the omentum or transverse colon or moving the camera port cephalad from the umbilicus. Once exposed, Calot's triangle was fully dissected to expose the arterial and biliary structures. If this area could not be exposed adequately or dissected properly then a retrograde or "fundus first" dissection was carried out. Sharp dissection using electrocautery was used initially, however near the neck of the gallbladder blunt and hydro-dissection were used to expose the cystic artery and bile duct. If the liver could not be retracted safely by a simple grasping instrument then a fixed liver retractor was inserted (angled triangular Diamond-Flex liver retractor, Surgical Innovations Group, Leeds, England http://www.sigroupplc.com. Elemental Healthcare, Berkshire, England, http://www.elementalhealthcare.co.uk).

Advice was sought from the Research Ethics Committee of North Bristol Trust regarding this project and it was felt that as it was service evaluation/audit it did not require formal approval by the Committee. Patients gave consent for their details and images to be used.

A literature search was carried out using the key words "laparoscopic cholecystectomy, retrograde, fundus first, Mirizzi syndrome and laparoscopic liver retraction" on the PubMed (National Library of Medicine, http://www.ncbi.nlm.nih.gov/PubMed) online database. Cross references from the reference lists of articles obtained were also reviewed and the pertinent articles are discussed.

## Results

Of the 1041 LCs, 148 (14%) were emergency operations and there were 131 (13%) associated laparoscopic bile duct explorations. There were no bile duct injuries but conversion to open operation was required in six patients (0.6%). In addition, LC was abandoned in 2 elderly patients; both had very fibrous, contracted gallbladders, one with coexisting incidental cirrhosis. Both had been on the waiting list for many months with resolution of their symptoms and in 3 years of follow up they have not become symptomatic or required reoperation. There was one mortality in an 80 year old man who was moribund from perforated acute cholecystitis who underwent an emergency LC but died soon afterwards of overwhelming sepsis.

The cases and reasons for conversion are listed below:

1) Unsuspected Mirizzi syndrome (type II) with conversion to deal with the defect in the bile duct.

2) Abnormal anatomy with short cystic duct entering right hepatic duct. Conversion due to suspected bile duct injury which turned out not to be the case after more complete dissection.

3) Failed laparoscopic bile duct exploration with impaction of the basket and stone during transcystic duct exploration.

4) Failed laparoscopic bile duct exploration-impacted large bile duct stone with stent *in situ *from 2 previous ERCPs (endoscopic retrograde cholangiography).

5) Severe acute cholecystitis with laparoscopically uncontrollable bleeding from an aberrant artery in the gallbladder bed (emergency case).

6) Severe acute on chronic cholecystitis with dense pericholecystic adhesions in a patient with severe learning difficulties and years of undiagnosed abdominal pain (emergency case).

In none of these cases was an attempt made to a RLC as the problem was not exposure or ability to dissect Calot's triangle. With hindsight in case number 2, releasing the fundic traction and using a liver retractor might have avoided conversion.

**Figure 3:**
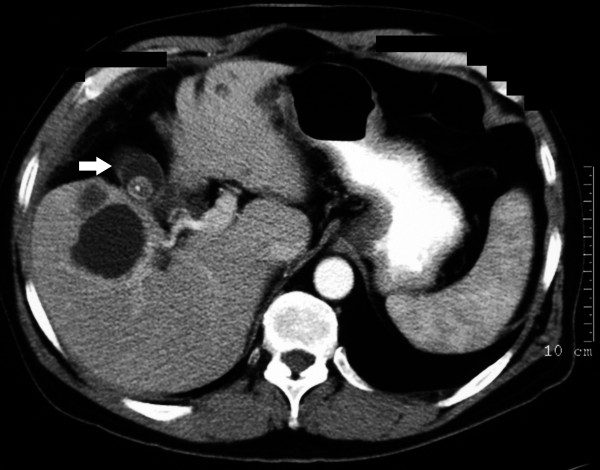
**Preoperative CT scan**. The scan shows a severely fibrotic, contracted gallbladder (arrow) and multiple simple liver cysts. Retrograde dissection was required in this patient (case 3) (Somatom Volume Zoom - 4 slice, Siemens AG, Erlangen, Germany).

**Figure 4:**
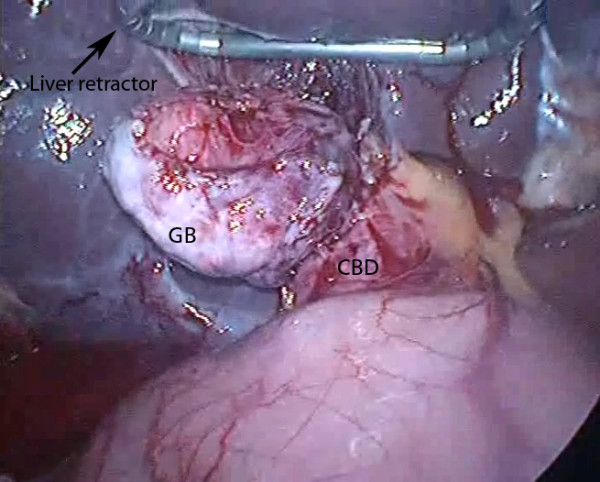
**Laparoscopic view of liver retraction**. The gallbladder (GB) has been mobilized prior to bile duct exploration via the large defect in the common bile duct (CBD) at its junction with the GB (case 5) (angled triangular Diamond-Flex liver retractor, Surgical Innovations Group, England http://www.sigroupplc.com. Elemental Healthcare, England, UK http://www.elementalhealthcare.co.uk).

**Figure 5:**
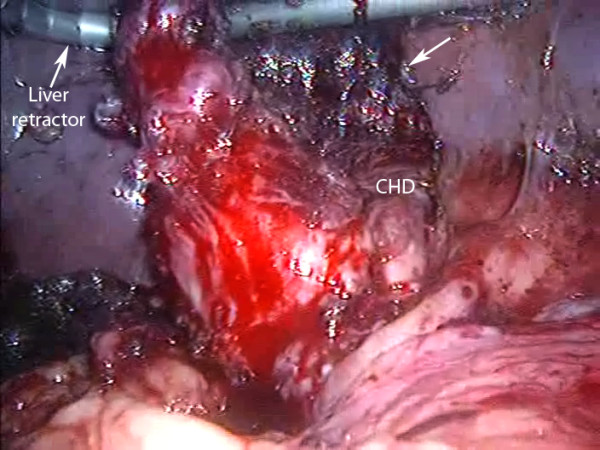
**Laparoscopic view showing liver retraction and retrograde dissection in acute Mirizzi syndrome**. Mobilisation of the inflamed and shrunken gallbladder was made possible by liver retraction (case 7). The arrow points to absorbable haemostatic gauze (surgicel, Ethicon, Somerville NJ, USA) in the gallbladder bed of the liver (CHD = common hepatic duct).

**Figure 6:**
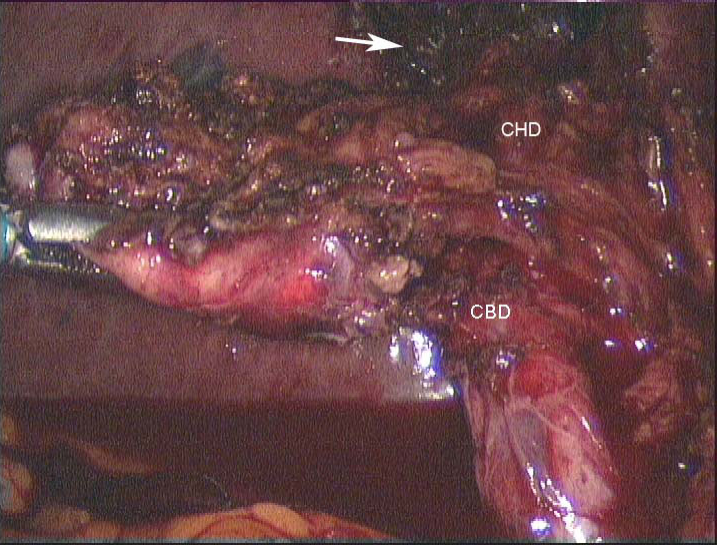
**Laparoscopic view of a mobilized, contracted gallbladder with a grasper retracting it laterally**. The anatomy is obvious now that the fundic traction has been relaxed and the GB freed from the liver, however the initial dissection was carried inadvertently to the medial side of the common bile duct (CBD) while there was strong cephalad fundic traction (case 9). The arrow points to surgicel in the gallbladder bed of the liver. (CHD = common hepatic duct).

**Figure 7:**
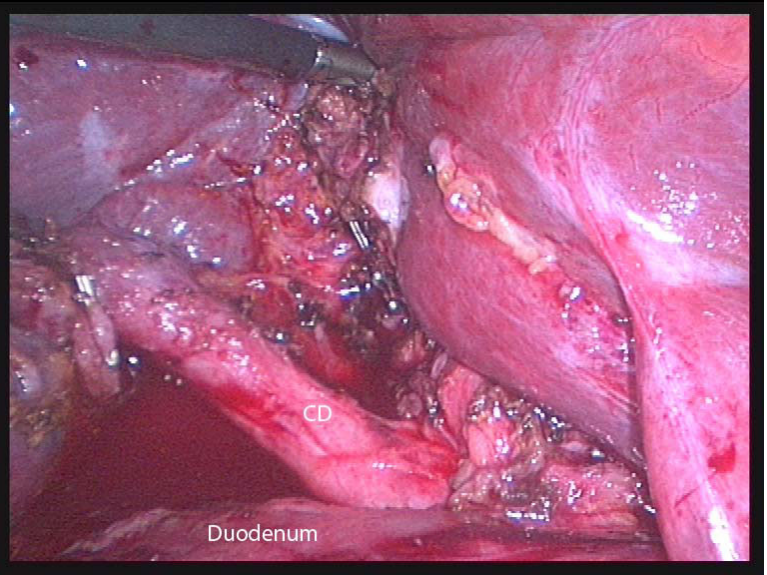
**Laparoscopic view of satisfactory retraction of the liver using a grasper**. There was a shrunken gallbladder around a cast of stones with aberrant cystic duct (CD) anatomy (case 10).

**Figure 8:**
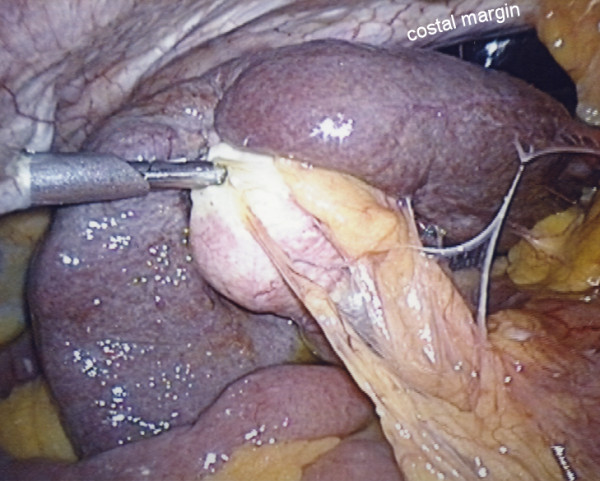
**Laparoscopic view showing the combination of kyphosis and a large, rigid right lobe of liver**. Antegrade dissection using fundic traction was not possible and "fundus first" dissection was needed (case 11).

RLC was attempted in 11 patients and successful in all (1.1%). The age ranged from 28 to 80 years (mean 61) and there were 7 males. In all cases it had not been possible using fundic traction to safely carry out the operation. The indications were severely fibrous, contracted gallbladder (GB) in 7 (coupled with aberrant anatomy in 1), Mirizzi syndrome in 2, severe kyphoscoliosis in 1 and severe kyphosis in 1. Postoperative stay was 1/2 to 5 days (mean 2.2) with no delayed sequelae on followup. The cases are described below and the histopathology was chronic cholecystitis unless stated;

1. A 42 year old man was admitted with jaundice and underwent ERCP with mechanical lithotripsy for a large bile duct stone. At subsequent LC there was a fibrous, contracted gallbladder and a fatty liver. (length of stay (LOS) 1 day) (figure 2).

2. A 54 year old man with a fibrous, contracted gallbladder (LOS 1).

3. A 60 year old man with a contracted gallbladder, which had been shown on preoperative CT scan (figure 3). Histopathology showed xanthogranulomatous inflammation and LOS was 1 day.

4. A 61 year old woman a fibrous, contracted gallbladder around a cast of stones. LOS was 5 days as she had to be reestablished on her warfarin therapy.

5. A 55 year old woman was admitted with jaundice and operated on as an emergency for a form of Mirizzi type II (figure 4). Choledochoscopy was needed for bile duct stones and the ductal defect was managed by a t-tube (LOS 3).

6. A 75 year old woman with severe kyphoscoliosis due to vertebral crush fractures from multiple myeloma was admitted as an emergency with gallstone pancreatitis and treated by ERCP. At subsequent LC standard fundic retraction failed to expose Calot's triangle due to her kyphoscoliosis and the lie of her liver (LOS 1).

7. A 70 yr old man, with jaundice, upper abdominal pain and fever. After failed ERCP for the Mirizzi syndrome, an emergency RLC was done (figure 5). Histopathology showed acute necrotising cholecystitis and the LOS was 4 days [8].

8. A 72 year old man with a fibrous contracted gallbladder. Histopathology showed xanthogranulomatous inflammation and LOS was 3 days.

9. A 69 year old man with an inflamed and fibrous, contracted gallbladder (LOS 2) (figure 6).

10. A 28 year old man with a gallbladder contracted around a cast of stones with a wide, long, parallel cystic duct. The anatomy was unclear until the fundus first dissection was complete (LOS 1/2 day) (figure 7).

11. An 80 year old woman with severe kyphosis was admitted as emergency with jaundice and pancreatitis. ERCP failed because of a pharyngeal pouch. LC and bile duct exploration was carried out. The liver retractor was tried but surprisingly it did not expose Calot's triangle as well as simple retraction with a grasper. Histopathology showed xanthogranulomatous inflammation and LOS was 3 days (figure 8).

Operative cholangiography was not used in these RLC cases. It would probably have been possible trancystic in 3 cases only (6, 10, 11) and after the gallbladder had been mobilized fundus first. In the other 8 cases, transcystic cholangiography would not have been possible due to fibrous obliteration as shown in figure 2 or Mirizzi syndrome (figure 5). Venous type bleeding from the gallbladder bed in the liver was controlled by pressure and absorbable haemostatic gauze. The cystic artery proper was often not clearly seen due to fibrosis and diathermy dissection close on the gallbladder wall. On several occasions moderate bleeding from the artery occurred near the neck of the gallbladder. This type of bleeding may be unsettling for an inexperienced laparoscopic surgeon however it was relatively straightforward to control using clips or diathermy. Management of the cystic duct was individualized and included use of titanium clips, intracorporeal suturing, endoloops and laparoscopic stapler (1 case). Drains were not routinely used. In two cases (10, 11) it was possible to maintain safe exposure using a simple grasper to elevate the liver (figure 7). In the remainder a liver retractor was needed and a fixed type was preferred to free up the assistant (figure 4, 5).

In most, but not all of the cases described above, there was preoperative suspicion that the operation would be difficult. From this series, predictors for the need for RLC included; Mirizzi syndrome (figure 5), severely contracted gallbladder on imaging (ultrasound or CT) (figure 3) or severe kyphosis. Three were operated on as emergencies for jaundice (27%) and 4 had undergone preoperative ERCP (36%) with failure in 2. There was xanthogranulomatous inflammation in 3 patients (27%).

## Discussion

French surgeons initially proposed laparoscopic "fundus first" dissection however it was the Reddick-Olsen technique of fundic traction to expose Calot's triangle, published in 1989, that became the standard technique worldwide [9]. Publications of the use of retrograde dissection of the gallbladder via laparoscopy began appearing in the mid 1990s. Kato et al dissected Calot's triangle first then took the gallbladder off the liver retrograde maintaining exposure by cephalad traction via a grasper on fundic serosa, which had been left attached to the liver [10,11]. Uyama *et al*. reported the feasibility of RLC maintaining exposure by suturing fundic serosa to the undersurface of the diaphragm [12]. Martin *et al. *reported use of a malleable laparoscopic liver retractor and noted that once the liver is retracted, dissection of the gallbladder can commence either at the fundus or at Calot's triangle [9]. If a liver retractor is used, safe antegrade dissection may be possible as sometimes it is the fundic traction, *per se*, on the contracted gallbladder that causes the problem with exposure and dissection. In some cases of Mirizzi syndrome, it is advantageous to mobilise the gallbladder from the liver first before dissecting near the CBD.

It is obviously simpler and quicker to use a grasper rather than a liver retractor. However, in the series detailed herein, use of a grasper to hold the liver up, although always tried initially, was only possible safely in two cases (figure 7) and this was more by pushing directly on the liver than by traction on fundic serosa left attached to the liver. In straightforward cases, grasping the fundic serosa should maintain exposure however most surgeons would not use fundus first dissection in these "easy" cases.

Various authors have confirmed the feasibility RLC in patients with acute or chronic inflammation and suggested it might decrease the rate of BDI [13-16]. In addition, several authors have reported that RLC helps to avoid open surgery. Mahmud et al reported that the use of fundus-first dissection in difficult cases decreased the conversion rate from a potential 5.2% to 1.2% [17]. Similarly Gupta *et al. *reported a decrease in conversion rate in a small series of patients with chronic cholecystitis from 18.8% to 2.1% [18]. Palanivelu *et al. *reported 265 LCs in cirrhotic patients and noted that liver retraction was needed in some cases to allow exposure of Calot's triangle and that RLC was resorted to in 8.3% of cases [19]. Ainslie et al. noted that liver retraction and RLC confers an advantage in difficult cholecystectomies because it opens the angle between the cystic duct and bile duct and contributed to their low conversion rate with no bile duct injuries [20]. Tuveri *et al. *reported a large series where RLC was used in 1.5% of cases due to difficult anatomy in Calot's triangle with a success rate of 80% [21].

Some authors have recommended routine use of RLC rather then reserving it for difficult cases. Cengiz *et al. *randomized 80 elective patients to compare the two dissection techniques and found that RLC combined with ultrasonic dissection was quicker and associated with less nausea and pain [22]. Ichihara et al reported tape ligature of the cystic duct then fundus first dissection in 500 patients and recommended it as a way of decreasing rates of BDI [23]. Yamakawa *et al. *described a case where they felt that RLC avoided a BDI in a patient with aberrant biliary anatomy [24]. Wang *et al. *presented a series showing that RLC was safe and effective in elderly patients with acute cholecystitis [25]. Neri *et al. *reported that RLC reduced the operative time and was an easier technique to perform [26]. They proposed that it should be the standard procedure and not only reserved for difficult cases.

However, the fact that most surgeons do not use RLC routinely shows that RLC is a more complex operation and is in keeping with the principle of Ockham's razor, that the simplest solution is the best. In the series detailed herein, RLC was resorted to only in difficult cases where standard technique had failed to provide adequate exposure or to allow safe dissection. It was not needed in cirrhotic patients (6 only in this series) or left sided gallbladder (1 case) [27]. Dolan *et al. *cautioned against RLC because they felt that the technique had caused a stone to be displaced into the bile duct in one patient [28]. In only 2 of the cases detailed herein (cases 6 and 10) was there an arrangement whereby this was a likely possibility (i.e. wide and patent cystic duct). Numerous series presented above quote high rates for operative cholangiography, for example 74% in Tuveri *et al*.'s report, even though they state that fundus first dissection was reserved for the very difficult cases [21]. If RLC is reserved for difficult cases as detailed herein then transcystic cholangiography will usually not be possible. If a RLC is carried out purely because of a problem with exposure (e.g. severe kyphoscoliosis) or identification (e.g. aberrant biliary anatomy) then transcystic cholangiography should usually be possible.

Full dissection of Calot's triangle with the neck of the gallbladder mobilized from the liver bed and operative cholangiography are recommended to avoid BDI [3]. Even in difficult cases such as Mirizzi syndrome, full Calot's triangle dissection may still be possible using standard technique. This is illustrated in figure 1 (a case from this series) where cephalad fundic traction allowed easy and safe dissection. However standard fundic traction sometimes fails to give adequate exposure when there is a contracted gallbladder (figure 2) and it would be accepted practice to convert to open surgery.

Conversion is not generally viewed as a complication and therefore most surgeons do not persist laparoscopically when the procedure is difficult. However, these cases are often just as difficult open, especially in obese patients, and BDIs do occur after conversion [29]. The magnified view at laparoscopy should be an advantage in the difficult LC so long as adequate exposure can be obtained. Obviously, if there is laparoscopically uncontrollable haemorrhage or an inability to expose the gallbladder or Calot's triangle then conversion must be carried out. In the present series, 2 cases were converted to open surgery for these reasons and the other 4 conversions were for bile duct problems including impacted stones or Mirizzi or suspected injury.

One other reason for conversion is that it allows the surgeon to use their sense of touch and this is generally accepted as a way to increase the safety of an operation especially one in which there is severe inflammation. Although some degree of tactility is possible via laparoscopic instruments (although not by currently available robotic systems) there is always going to be a case that has to be converted to allow proprioception or the touch of the surgeon's hand to increase patient safety. Hand-assisted LC has been proposed as a way to allow proprioception without open surgery [30].

Bleeding from the gallbladder bed of the liver tracking down and obscuring the view is a theoretical problem in RLC although it was not a significant problem in the current series. Extra care should be taken near the neck of the gallbladder as bleeding may occur from the cystic artery. The surgeon undertaking these difficult cases needs to be comfortable dealing with bleeding laparoscopically.

## Conclusions

Conversion is necessary when there is inability to expose the gallbladder, uncontrollable haemorrhage or problems with the bile duct that cannot be dealt with laparoscopically. In some LCs (1% in this series) standard technique (cephalad fundic traction and antegrade dissection) will fail and an alternative technique will be needed if conversion is to be avoided. A likely scenario whereby fundus first dissection would be needed is a combination of a contracted gallbladder, large liver and abnormal body habitus for example kyphoscoliosis. When fundus first dissection is reserved for difficult LCs (as would be the case for most surgeons) a laparoscopic liver retractor may be needed. RLC utilising a retractor does have a role and should be in the armamentarium of even experienced laparoscopic surgeons.

## Competing interests

The author declares that they have no competing interests.

## Pre-publication history

The pre-publication history for this paper can be accessed here:

http://www.biomedcentral.com/1471-2482/9/19/prepub

## References

[B1] McIntyre RC Jr Bensard DD Stiegman GV Pearlman NW Durham J Exposure for laparoscopic cholecystectomy dissection alters biliary ductal anatomy Surg Endosc 1996 10 41 3 10.1007/s004649910010 8711604

[B2] Hunter JG Avoidance of bile duct injury during laparoscopic cholecystectomy Am J Surg 1991 162 71 6 10.1016/0002-9610(91)90207-T 1829588

[B3] Strasberg SM Avoidance of biliary injury during laparoscopic cholecystectomy J Hepatobiliary Pancreat Surg 2002 9 543 547 10.1007/s005340200071 12541037

[B4] Hugh TB Kelly MD Mekisic A Rouviere's sulcus: A useful landmark in laparoscopic cholecystectomy Br J Surg 1997 84 9 1253 1254 10.1002/bjs.1800840916 9313706

[B5] Hugh TB New strategies to prevent laparoscopic bile duct injury-surgeons can learn from pilots Surgery 2002 132 826 35 10.1067/msy.2002.127681 12464867

[B6] De Pouvourville G Ribet-Reinhart N Fendrick M Houry S Testas P Huguier M A prospective comparison of the costs and morbidity of laparoscopic versus open cholecystectomy Hepatogastroenterology 1997 44 35 9 9058115

[B7] Jenkins PJ Paterson HM Parks RW Garden OJ Open cholecystectomy in the laparoscopic era Br J Surg 2007 94 1382 5 10.1002/bjs.5854 17654611

[B8] Kelly MD Acute Mirizzi syndrome JSLS 2009 13 104 9 19366554 PMC3015902

[B9] Martin IG Dexter Sp Marton J Gibson J Asker J Firullo A Fundus-first laparoscopic cholecystectomy Surg Endosc 1995 9 203 206 10.1007/BF00191967 7597594

[B10] Kato K Matsuda M Onodera K Kobayashi T Kasai S Mito M Laparoscopic cholecystectomy from fundus downward Surg Laparosc Endosc 1994 4 373 4 10.1097/00019509-199410000-00012 8000639

[B11] Kato K Kasai S Matsuda M Onodera K Kato J Imai M A new technique for laparoscopic cholecystectomy-retrograde laparoscopic cholecystectomy: an analysis of 81 cases Endoscopy 1996 28 4 356 359 8813502 10.1055/s-2007-1005480

[B12] Uyama I Iida S Ogiwara H Takahara T Kato Y Furuta T Laparoscopic retrograde cholecystectomy (from fundus downward) facilitated by lifting the liver bed up to the diaphragm for inflammatory gallbladder Surg Laparosc Endosc 1995 5 431 436 8611987

[B13] Raj PK Castillo G Urban L Laparoscopic cholecystectomy: fundus-down approach J Laparoendosc Adv Surg Tech A 2001 11 2 95 100 11327135 10.1089/109264201750162374

[B14] Rosenberg J Leinskold T Dome down laparoscopic cholecystectomy Scand J Surg 2004 93 48 51 15116820 10.1177/145749690409300110

[B15] Sekimoto M Tomita N Tamura S Ohsato H Monden M New retraction technique to allow better visualization of the Calot's triangle during laparoscopic cholecystectomy Surg Endosc 1998 12 1439 41 10.1007/s004649900877 9822475

[B16] Ota A Kano N Kusanagi H Yamada S Garg A Techniques for difficult cases of laparoscopic cholecystectomy 2003 10 172 5 14505152 10.1007/s00534-002-0825-4

[B17] Mahmud S Masaud M Canna K Nassar AHM Fundus-first laparoscopic cholecystectomy Surg Endosc 2002 16 581 584 10.1007/s00464-001-9094-6 11972192

[B18] Gupta A Agarwal PN Kant R Malik V Evaluation of Fundus-First Laparoscopic Cholecystectomy JSLS 2004 8 3 255 258 15347114 PMC3016813

[B19] Pananivelu C Rajan PS Jani K Shetty AR Sendhilkumar K Senthilnathan P Laparoscopic cholecystectomy in cirrhotic patients: the role of subtotal cholecystectomy and its variants J Am Coll Surg 2006 203 2 145 151 16864026 10.1016/j.jamcollsurg.2006.04.019

[B20] Ainslie WG Larvin M Martin IG McMahon MJ Liver retraction techniques for laparoscopic cholecystectomy Surg Endosc 2000 14 311 10.1007/s004640000063 28337612

[B21] Tuveri M Calo PG Medas F Tuveri A Nicolosi A Limits and advantages of fundus-first laparoscopic cholecystectomy: lessons learned 2008 18 69 75 18266578 10.1089/lap.2006.0194

[B22] Cengiz Y Janes A Grehn A Israelson LA Randomized clinical trial of traditional dissection with electrocautery *versus *ultrasonic fundus-first dissection in laparoscopic cholecystectomy Br J Surg 2005 92 7 810 813 15880649 10.1002/bjs.4982

[B23] Ichihara T Takada M Ajiki T Fukumoto S Urakawa T Nagahata Y Tape ligature of cystic duct and fundus-down approach for safety laparoscopic cholecystectomy: Outcome of 500 patients Hepatogastroenterology 2004 51 362 364 15086159

[B24] Yamakawa T Zhang T Midorikawa Y Ishiyama K Sugiyama Y A case of cystic duct drainage into the left intrahepatic duct and the importance of laparoscopic fundus-first cholecystectomy for the prevention of bile duct injury J Laparoendosc Adv Surg Tech A 2007 17 5 662 665 17907984 10.1089/lap.2006.0240

[B25] Wang Y-C Yang H-R Chung P-K Jeng L-B Chen R-J Role of fundus-first cholecystectomy in the management of acute cholecystitis in elderly patients J Laparoendosc Adv Surg Tech A 2006 16 2 124 127 16646701 10.1089/lap.2006.16.124

[B26] Neri V Ambrosi A Fersini A Tartaglia N Valentino TP Antegrade dissection in laparoscopic cholecystectomy JSLS 2007 11 225 8 17761085 PMC3015719

[B27] Kelly MD Craik JD Left side gallbladder revisited ANZ J Surg 2008 78 192 3 10.1111/j.1445-2197.2007.04401.x 18269487

[B28] Dolan JP Cook JW Sheppard BC Case report: Retained common bile duct stone as a consequence of a fundus-first laparoscopic cholecystectomy J Laparoendosc Adv Surg Tech A 2005 15 3 318 321 15954837 10.1089/lap.2005.15.318

[B29] Hugh TB Laparoscopic bile duct injury: Some myths ANZ J Surg 2002 72 164 167 10.1046/j.1445-2197.2002.02311.x 12074074

[B30] Wei Q Shen LG Zheng HM Hand-assisted laparoscopic surgery for complex gallstone disease World J Gastroenterol 2005 11 21 3311 3314 15929191 10.3748/wjg.v11.i21.3311 PMC4316072

